# Managing appendicitis during the COVID-19 era: A single centre experience & implications for future practice

**DOI:** 10.1016/j.amsu.2021.02.014

**Published:** 2021-02-12

**Authors:** Oreoluwa Bajomo, Rumneek Hampal, Paul Sykes, Anur Miah

**Affiliations:** a Royal Liverpool University Trust Hospital, Prescot Street, Liverpool, L7 8XP, UK; b Hospital: Kingston Hospital, NHS Foundation Trust, UK; c Portsmouth Hospitals NHS Trust, UK; d Medway Maritime Hospital, Windmill Way, Gillingham, Kent, ME7 5NY, UK

**Keywords:** COVID-19, Appendicitis, Pandemic, Management, Case series

## Abstract

**Aim:**

During the COVID-19 pandemic, emergency surgery was modified in line with Royal College guidance to accommodate the evolving climate. This study compared management of appendicitis before and during the pandemic by assessing disease presentation severity, modes of investigation, surgical management and patient outcomes. Outcomes assessed included length of stay, readmissions and rates of postoperative wound infections.

**Methods:**

We collected data on appendicitis patients managed at a district general hospital over two distinct 8-week periods; 42 patients before and 36 patients during the COVID-19 pandemic respectively. The study included clinically or radiologically diagnosed appendicitis patients.

**Results:**

Our study found patients during the COVID-19 pandemic had higher inflammatory markers (CRP 103 vs 53 mg/L; p = 0.03) and more severe disease on histological examination of the appendix than pre-pandemic. Patients were nearly twice as likely to undergo CT diagnosis of appendicitis during the pandemic than before. During the pandemic, only half of the cohort underwent laparoscopic appendicectomy in contrast with greater than 85% of the pre-COVID-19 cohort (p = 0.0005). Patients in the COVID-19 era cohort recorded shorter lengths of hospital stay (2.6 vs 3 days; p = 0.35); however, had higher reattendance rates (12 vs 25%; p = 0.15) and surgical site infections (p = 0.0443). Finally, the study reported shorter median time to theatre (0 vs 1 days) during the pandemic than before.

**Conclusion:**

In addition to reiterating the benefits of laparoscopic versus open surgery and quicker diagnostic methods, this study also implies that though patients during COVID-19 era presented with more severe disease, their treatment was in a more efficient service.

## Highlights

• Patients presented with more severe cases of appendicitis during the COVID-19 pandemic.
• A higher proportion of patients with suspected appendicitis underwent CT imaging during the pandemic.
• Appendicitis patients were more likely to be managed conservatively during the COVID pandemic.
• Surgically managed patients were more likely to undergo open surgery during the pandemic.
• Patients had shorter lengths of stay but higher chances of post-operative complications during the pandemic.

## 1 Introduction

The SARS-CoV-2 Coronavirus (COVID-19) pandemic has challenged healthcare systems and institutions worldwide in ways unexpected, with direct and indirect consequences to the delivery to patient care. Efforts to manage COVID-19 have included capacity-building strategies such as suspension of non-essential hospital services and implementation of new healthcare delivery pathways. At the height of the pandemic, surgical services offered in the United Kingdom were limited to life or limb threatening conditions, performed under unique hospital guidelines to minimize physician and patient exposure to COVID-19.

Accordingly, The Royal College of Surgeons released a timely document proposing nationwide guidelines on operating during the pandemic [1]. The document largely opposed laparoscopic surgery in response to studies, which reported the possibility of viral shedding within peritoneal fluid [2]. Open surgical procedures were encouraged with appropriate personal protective equipment (PPE) advised and radiological confirmation of diagnosis encouraged prior to surgery [1]. As such, operations such as appendicectomies, which are now predominantly performed laparoscopically, witnessed a resurgence of historic complications from open procedures. We present our experience of managing appendicitis patients at a hospital in the UK during the COVID-19 pandemic. This study aims to compare changes in practice to appendicitis management triggered by the COVID-19 pandemic by comparing clinical presentation, investigation modality, treatment strategies and outcomes before and during the pandemic.

## 2 Methods

We conducted a case series on data retrieved from a prospectively maintained inpatient surgical database (PILES) and compared patients who presented with appendicitis during the COVID-19 pandemic era with patients who presented before the COVID-19 pandemic. Data was collated on patients in the pre-COVID-19 era over a 2-month period from 14th January - 14th March whilst data from the COVID-19 era dated between 14th March and 14th May at a single centre district general hospital. Follow-up data including postoperative complication data was retrieved from hospital records on re-attending patients following discharge. Data was analyzed using GraphPad Prism 8 and non-parametric statistical methods. All patients diagnosed with appendicitis either clinically or radiologically during specified time windows were included in the study and duplicate records were excluded. We also excluded patients who were clinically coded as appendicitis but did not undergo an operation due to clinical or radiological exclusion of appendicitis. Due to the emergency nature of appendicitis presentation, patients did not routinely require pre-intervention optimization This study was registered at Research Registry with unique ID: researchregistry6347. ‘This case series has been reported in line with the PROCESS 2018 criteria [11].

## 3 Results

### 3.1 Comparison of patient demographics of pre-COVID-19 and COVID-19 era cohorts

**Fig. 1:**
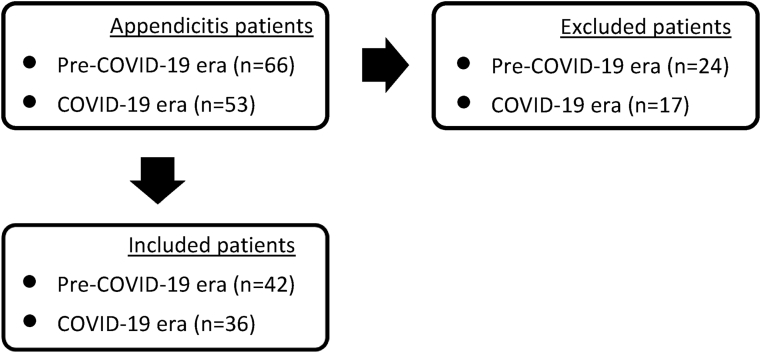
Flowchart demonstrating patient selection process.

We identified 42 patients who presented with acute appendicitis in the pre-COVID-19 data collection period and 36 patients during the COVID-19 data collection period. 61.9% patients who presented prior to the onset of the pandemic were male with an average age of 30.4 years (range 7–83 years). 55.6% of patients who presented during the pandemic were male with a mean age of 28 years (range 6–92 years) (p = 0.655) (see Fig. 1) (Table 1).

**Table 1:** Summary of patient characteristics included in the study.

	Pre-COVID-19 cohort	COVID-19 cohort	p value
Gender			
Male % (n)	61.9(26)	55.6 (20)	
Female % (n)	38.1(16)	44.4 (16)	
Mean age (years)	30.5	26.5	0.6554
Serum white cell count (x10^9^/L)	12.4	13.5	0.1326
Serum C-reactive protein (mg/L)			
Surgical management % (n)	53.4	103.2	0.03*
Histology	100 (42)	86 (31)	0.0179*
Advanced disease	21 (9)	29 (9)	0.5842
Normal	9.5 (4)	0 (0)	0.1317
Imaging prior to operation			
CT imaging % (n)	38.1 (16)	64.5 (20)	0.0340*
US imaging % (n)	11.9 (5)	12.9 (4)	>0.999
No imaging % (n)	50 (21)	22.6 (7)	0.0277*

* - Statistically significant p-values.

### 3.2 Severity of appendicitis

**Fig. 2:**
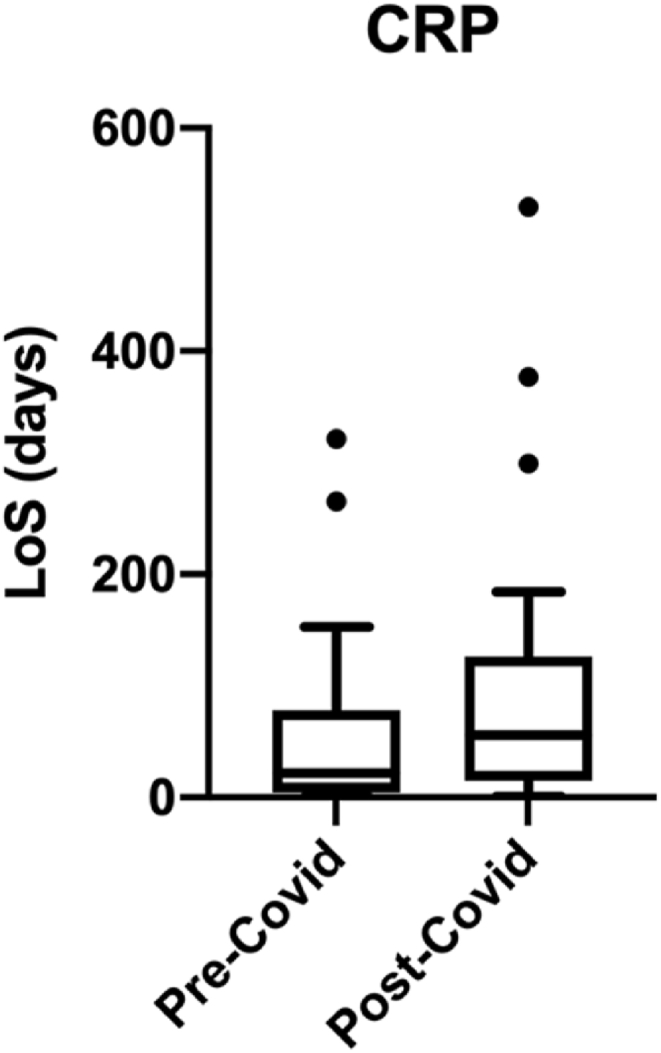
Comparison of C-reactive protein in patients diagnosed with appendicitis before and during the COVID-19 pandemic.

**Fig. 3:**
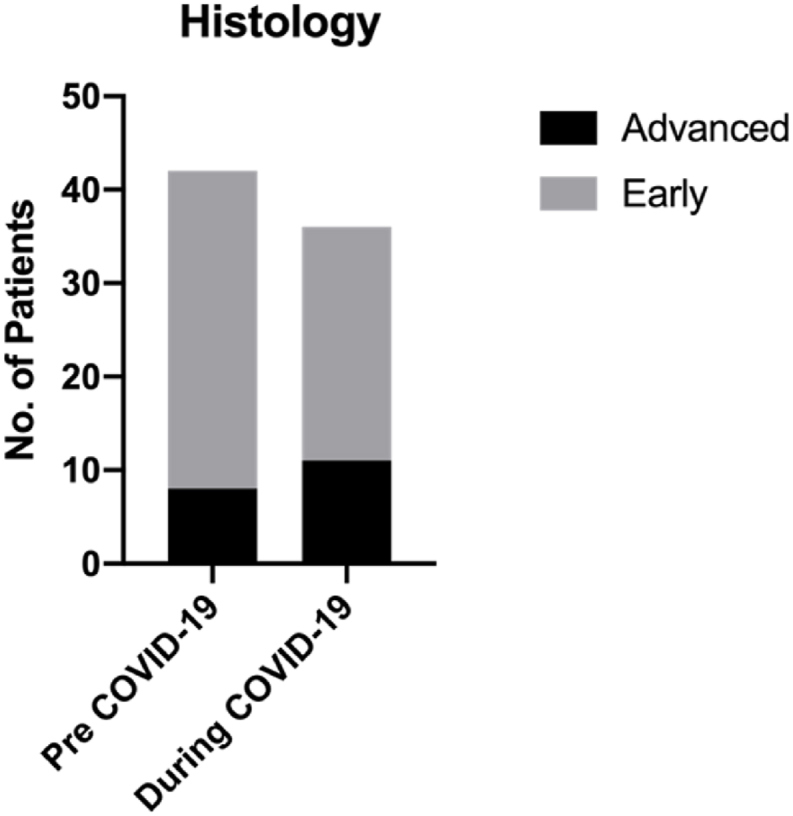
Comparison of appendix histology before and during the COVID-19 pandemic.

Patients diagnosed with appendicitis during the designated COVID-19 period were found to have a greater degree of inflammation as evidenced by higher serum acute phase proteins and more severe histological disease staging. During the pandemic, patients were noted to have a slightly higher mean serum white cell count (WCC) of 13.5 × 10^9^/L than patients who presented prior to the pandemic with an average serum WCC of 12.4 × 10^9^/L (p = 0.1326). However, patients demonstrated significantly higher mean C-reactive protein (CRP) of 103 mg/L during the pandemic than prior with an average CRP of 54.0 mg/L (p = 0.03) (Fig. 2). Furthermore, histological analysis post-operatively demonstrated a third of all appendixes removed (9 out of 31) during the COVID-19 timeframe showed advanced disease either with perforation or gangrene, compared with 9 out of 42 appendixes with advanced pathology (20%) prior to the pandemic (p = 0.5842) (Fig. 3). Histological evidence of perforation or gangrene was classified as advanced disease whilst inflammation alone was considered non-advanced.

Whilst one out of every 10 appendicectomies performed before the pandemic revealed normal histology, all appendicectomies performed during the COVID-19 era showed histological markings of appendicitis (p = 0.1317). Interestingly, a further 10% of appendicectomies prior to the pandemic unusually had various forms of enterobius vermicularis on histology, none of such cases were detected during the pandemic. Before and during the COVID-19 pandemic,14% of cases showed moderate disease pathology with appendiceal suppuration found on histological examination.

### 3.3 Investigation of acute appendicitis prior to and during COVID-19 delay phase

**Fig. 4:**
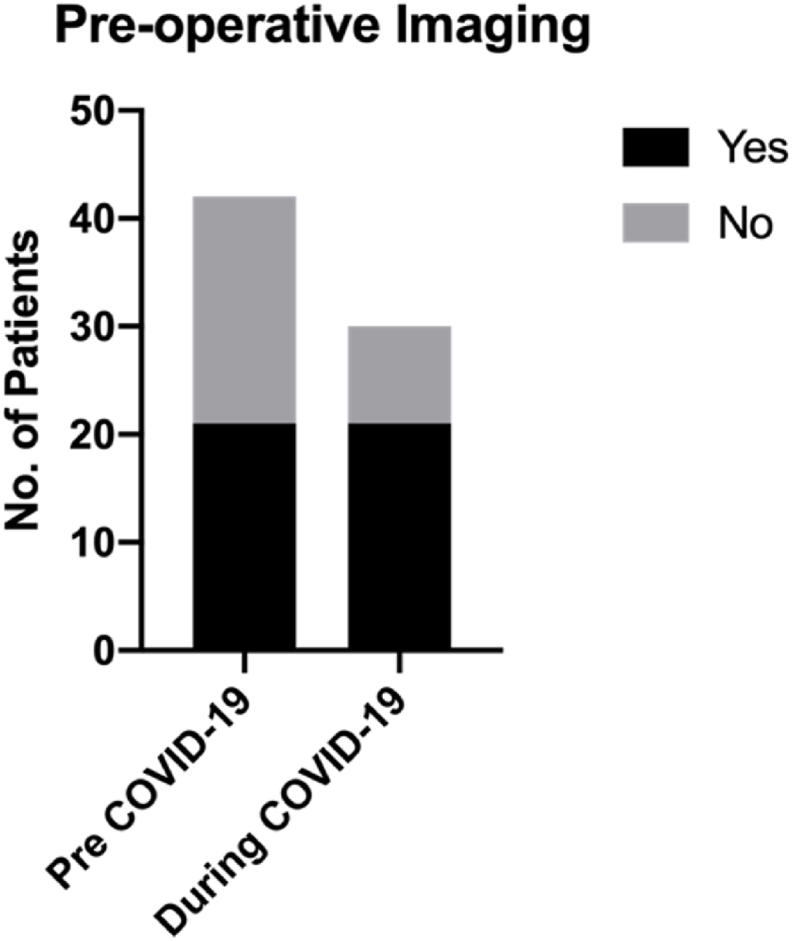
Comparison of patients undergoing pre-operative CT imaging before and during the COVID-19 pandemic.

**Fig. 5:**
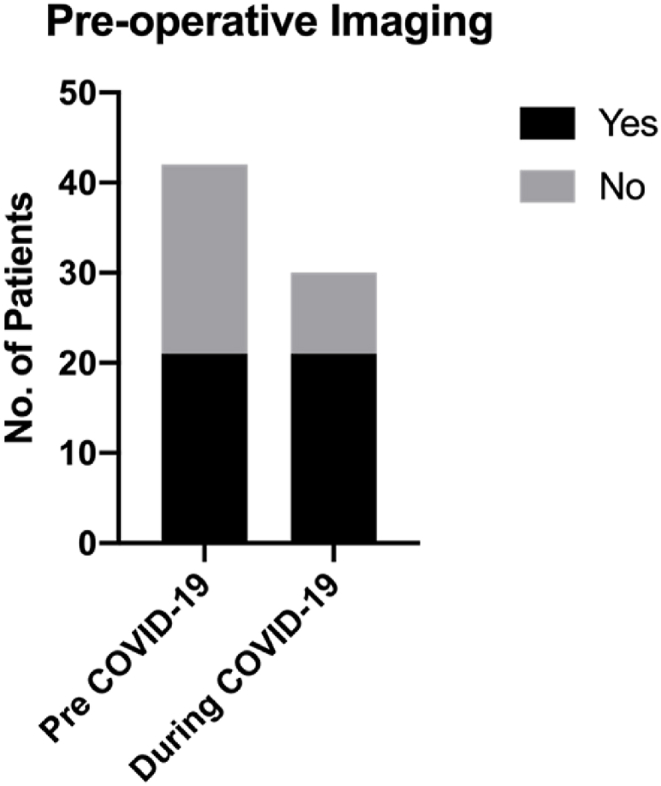
Comparison of patients who did not undergo imaging before and during the COVID-19 pandemic.

Imaging modalities utilized were analyzed to determine adherence to recommendations made by national bodies at the onset of the pandemic; the use of computed tomography (CT) in particular was advised to confirm pathology prior to surgical intervention. Prior to the COVID-19 pandemic, 38% underwent CT prior to surgery whilst 50% of the cohort was diagnosed clinically and underwent surgery without radiological evidence (Fig. 4) (Fig. 5). During the COVID-19 period 65% (p = 0.034) of appendicitis patients underwent pre-operative CT imaging to confirm their diagnosis in line with College guidance, 13% underwent ultrasound imaging while 23% (p = 0.0277) of appendicitis patients did not undergo any radiological investigation before their operation.

A higher proportion of chest radiographs were performed on patients during the pandemic (8 patients) than was noted prior to the pandemic (4 patients) as this as used to screen patients for concurrent coronavirus infection.

### 3.4 Management of acute appendicitis prior to and during COVID-19

**Fig. 6:**
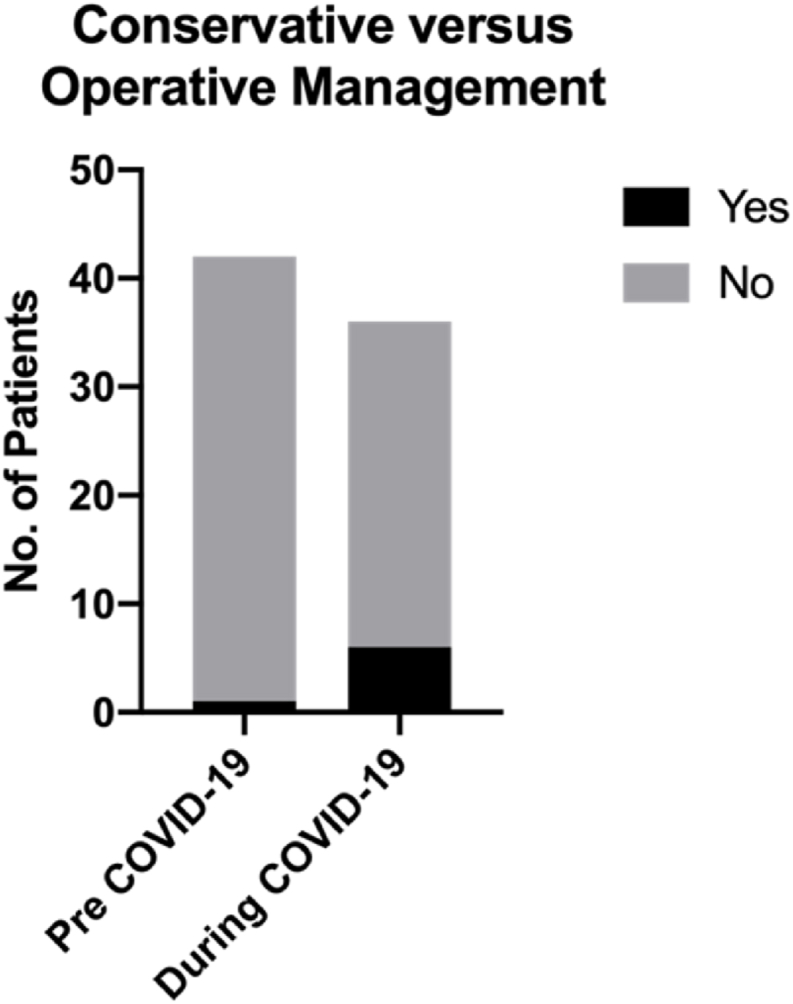
Comparison of conservative versus surgical management before and during the COVID-19 pandemic.

Pre-COVID-19, acute appendicitis was primarily managed surgically in all 42 documented cases, however during the COVID-19 pandemic, 5 out of 36 identified appendicitis patients were managed conservatively with antibiotics alone (p = 0.0179) (Fig. 6). Antibiotics (Co- amoxiclav or ciprofloxacin for penicillin allergy) were prescribed according to local hospital guidelines and remained unchanged before and during the pandemic.

**Fig. 7:**
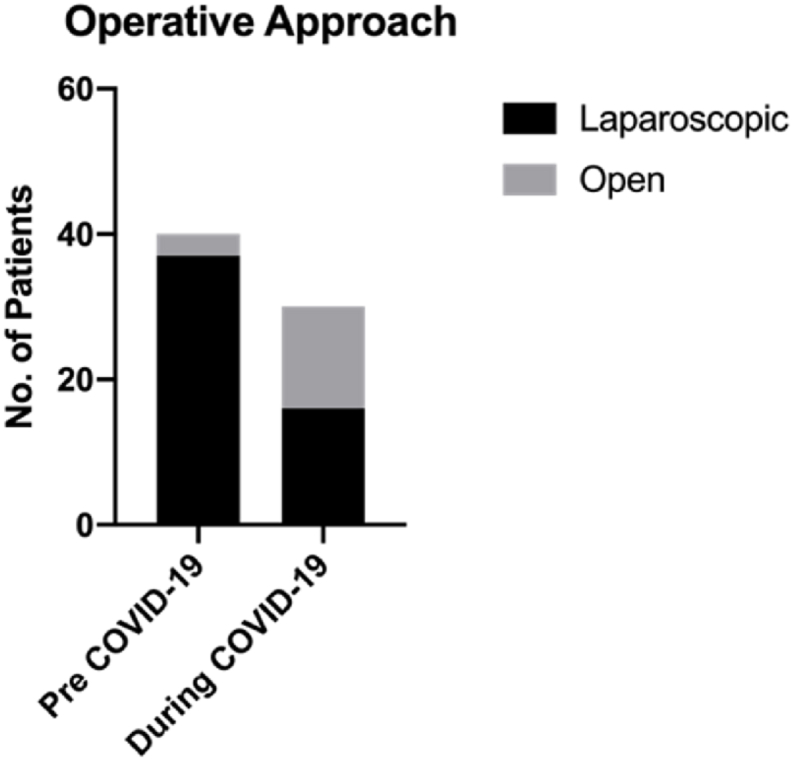
Comparison of laparoscopic versus open surgical approach before and during the COVID-19 pandemic.

Prior to COVID-19, 37 out of 42 appendicitis patients were managed laparoscopically whilst there was increased number of open procedures performed during the pandemic (16/31) (p = 0.0005) (Fig. 7). Operations were performed by a mis of Consultants and junior doctors in both groups.

**Fig. 8:**
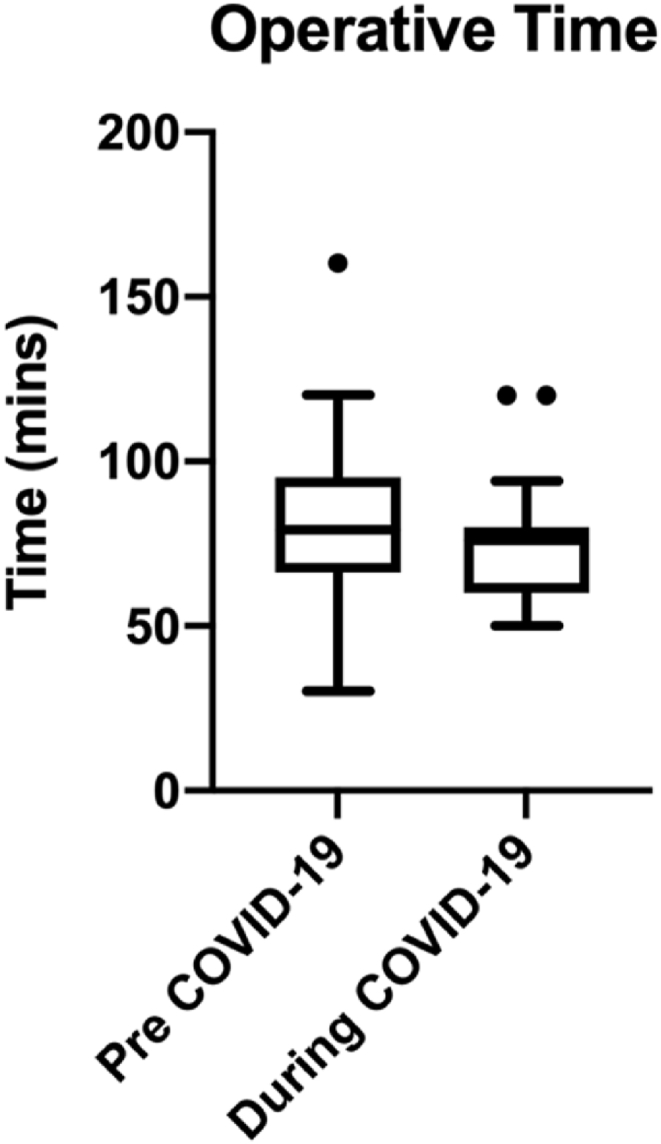
Comparison of operative time before and during the COVID-19 pandemic.

**Fig. 9:**
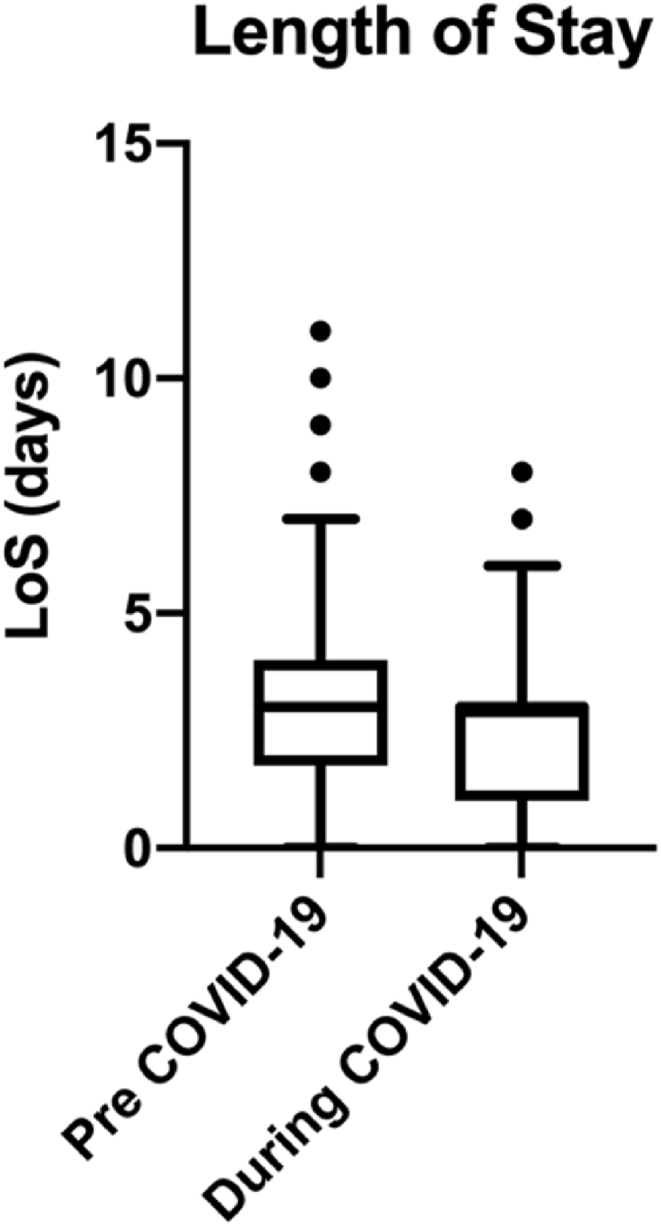
Comparison of length of stay (LoS) before and during the COVID-19 pandemic.

The median time from presentation to theatre was 1-day pre-COVID-19 with an average operative duration of 80.6 min. During COVID-19 the median time from presentation to theatre was shorter at 0 days and operation time also reduced, to an average 73.0 min (p = 0.26) (Fig. 8). Despite presenting with more severe disease, patients required shorter average lengths of stay (LOS) in hospital during the COVID-19 pandemic - 2.6 days (range 0–8 days) than prior to the pandemic, 3.0 days (range 0–13 days) (p = 0.35) (Fig. 9).

### 3.5 Complications in patients with acute appendicitis

**Fig. 10:**
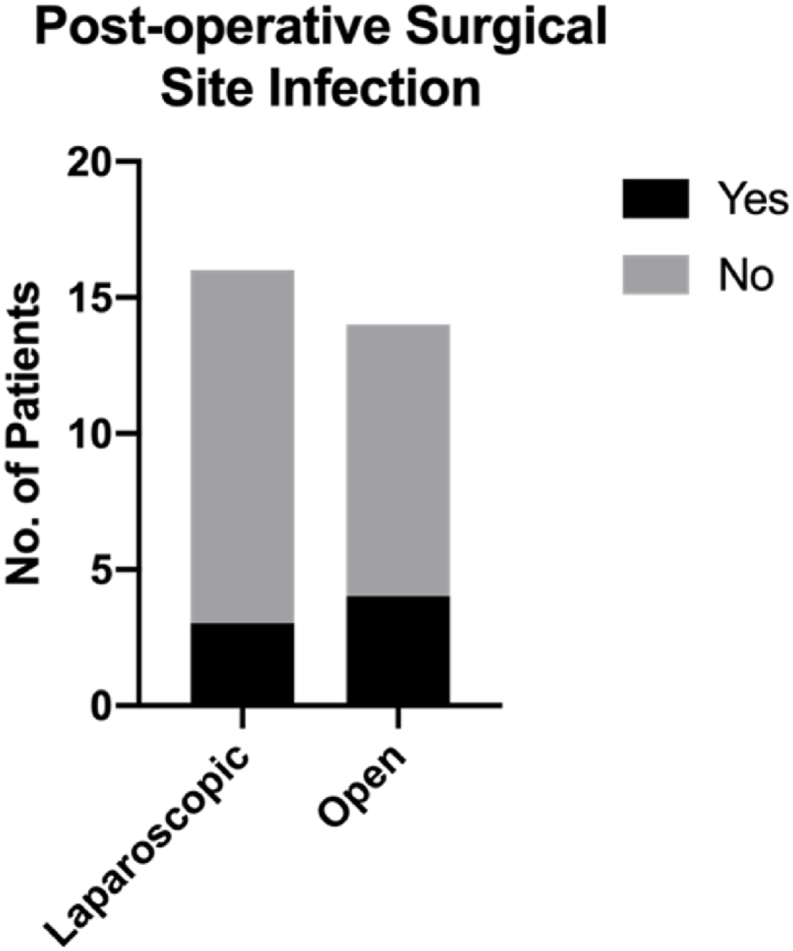
Comparison of postoperative complications in laparoscopic versus open approach within the COVID cohort.

In the COVID-19 era group, 9 patients re-presented to hospital following the development of post-operative complications (Fig. 10). One patient presented with COVID symptoms, two patients with conservatively managed postoperative abdominal pain (Clavien Dindo grade I) whilst three had wound infections only (2 Clavien Dindo I, 1 Clavien Dindo IIIb). The other 3 patients presented with intra-abdominal collections – 1 was conservatively managed (Clavien Dindo I) and the final two patients required laparotomy (Clavien Dindo IIIb)

**Fig. 11:**
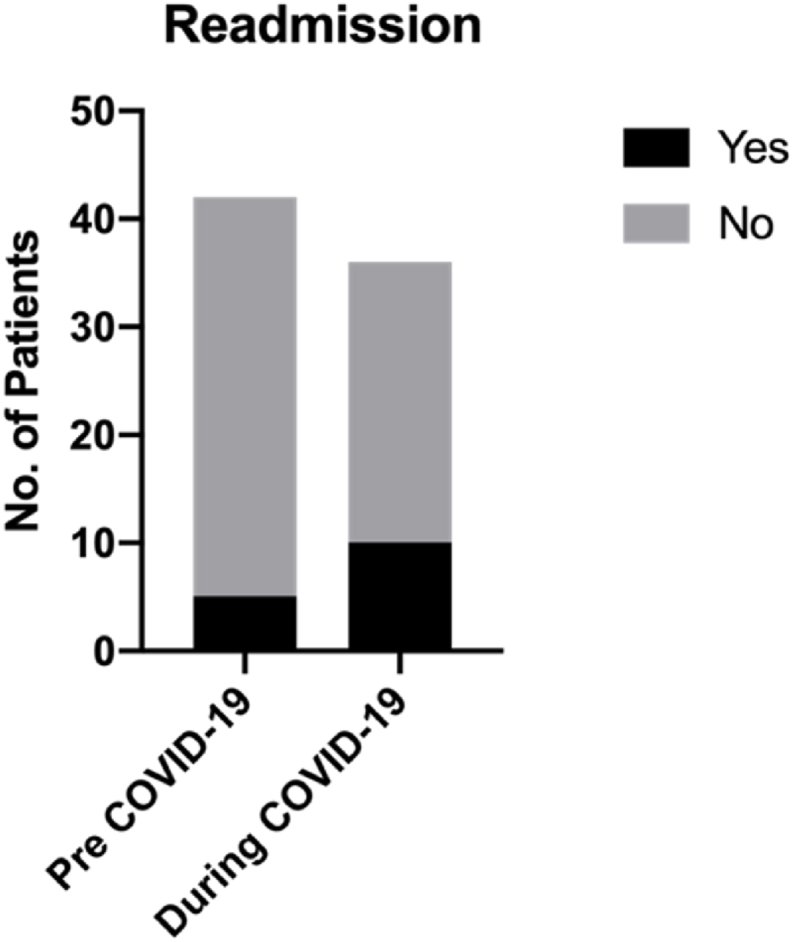
Comparison of postoperative hospital re-attendance before and during the COVID-19 pandemic.

Specifically, within the COVID-19 era cohort, only 3 out of 15 laparoscopically managed cases developed complications while 6 out of 16 open cases developed complications (Fig. 11) (p = 0.4331).

In the pre-COVID-19 cohort, 5 patients represented to hospital – 4 of which complained of conservatively managed postoperative abdominal pain (Clavien Dindo 1) and one patient suffered a post-operative collection which required operative drainage (Clavien Dindo IIIb).

**Fig. 12:**
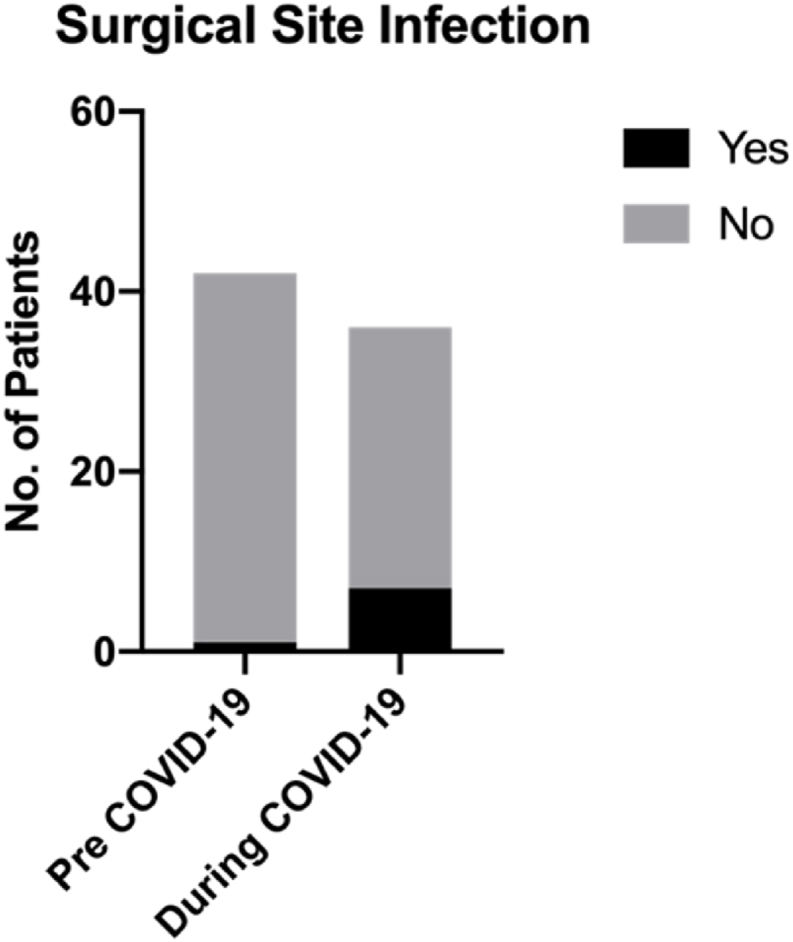
Comparison of surgical site infections before and during the COVID-19 pandemic.

With regards to surgical site infections, patients in the COVID-19 era cohort suffered significantly higher proportion of infections than pre-COVID (p = 0.043) (Fig. 12).

## 4 Discussion

Acute appendicitis is a common surgical emergency presentation, which often presents acutely with right-sided lower abdominal pain associated with nausea and/or vomiting and raised inflammatory markers. The incidence of appendicitis in the United Kingdom is approximately 1 in 400 [3], with 50,000 appendicectomies performed annually[4]. Whilst our study did not find any significant difference in the number of appendicitis presentations, we did however note more advanced disease pathology during the pandemic than before. Although not assessed in this study, we believe more advanced disease presentation occurred due to longer delays in emergency general surgical presentation associated with COVID-19 anxiety [5]. Specific to appendicitis, delayed presentation often leads to worsened clinical condition with an increased likelihood for appendiceal perforation and resulting intra-abdominal contamination. Our study confirms this phenomenon with higher admission inflammatory markers and a greater proportion of resected appendices having histological confirmation of gangrene or perforation during the COVID-19 pandemic than before.

In keeping with national practice, our study found pre-pandemic diagnosis of appendicitis to rely heavily on clinical examination - 50% of the cohort was operated upon based on clinical history, examination findings and blood parameters with an overall 10% negative appendicectomy rate. During the pandemic however, advice to acquire CT imaging in diagnosing appendicitis resulted in more rapid confirmation of disease and a 100% positive appendicectomy rate. Quicker confirmation of diagnosis during the pandemic also resulted in shorter time to theatre and subsequently reduced length of stay in spite of more advanced disease pathology. This demonstrates a significant change in our clinical practice driven by the COVID-19 pandemic whereby appendicitis diagnosis required radiological confirmation due to the perceived risks of operating on patients with potential concurrent coronavirus illness. In addition to patient mortality risk estimated as nearly 25%, there also were added risks of transmission of COVID-19 to healthcare workers from anaesthetic and surgical aerosol generating procedures, hence the need for more accurate pre-operative diagnosis [6]. Though these measures were introduced to minimize unnecessary operations in high-risk COVID-19 environments, they have also demonstrated benefits of quicker appendicitis diagnosis which can be implemented in future practice.

The standard management of appendicitis is surgical removal of the appendix, with a laparoscopic approach considered gold standard[7]. It is widely acknowledged that laparoscopic surgery has several advantages over the open approach namely reduced LOS and reduced rates of post-operative complications such as wound infection and intra-abdominal collections [8,9]. During the pandemic, College advice to perform open instead of laparoscopic surgery was recommended to limit healthcare workers’ exposure to COVID 19 virus particles present in peritoneal fluid and aerosolized through pneumoperitoneum release [10]. In keeping with existing literature, our study consequently found an increased occurrence of post-operative complications in patients who attended during the pandemic and were consequently more likely to undergo open surgery [8,9]. Higher SSI rates are a likely combination of delayed presentation, shorter courses of intravenous antibiotics driven by shorter in-hospital stays as well as limitations of the open appendicectomy technique. Intra-operatively, insufflation of the abdomen during laparoscopic surgery offers superior views of the abdomen and greater effectiveness of intra-abdominal lavage.

During the COVID-19 era, shorter lengths of stay were noted despite more severe disease presentation and a preference for open rather than laparoscopic appendicectomies. This may reflect both patient and clinician concerns regarding potential nosocomial transmission of COVID-19 resulting in expedited discharge from hospital with emphasis on considered safety netting. In addition, reduced demand on surgical services may have also contributed to shorter delays to imaging and theatre thereby facilitating the discharge process. We also note that more severe disease presentation during the pandemic may have afforded greater NCEPOD priority resulting in shorter delays to theatre. Finally, hospital guidelines mandated the presence of a senior surgeon in operations performed during the pandemic which not only contributed to shorter operative time but may have even reduced the true extent of postoperative complications.

## 5 Conclusion

This study has highlighted inefficiencies in pre-COVID-19 appendicitis management such as prolonged LOS and decision to operate as a direct result of delays to radiological investigation. In spite of limitations such as small sample size, it has also re-emphasized the value of early presentation and appropriate investigation in appendicitis patient outcomes. We also note challenges in follow-up methodology, as patients who suffered complications but did not reattend may have been missed. We overcame the follow up challenges by extensively searching electronic health records however note a future similar study might include more structured prospective follow up planning. Finally, we prove that the laparoscopic approach has better outcomes than open appendicectomies and suggest that future national health emergency responses take existing literature into greater consideration. Future research endeavours in this topic area may include a systematic review of similar studies.

## Ethical approval

Ethical approval not required.

## Funding

None.

## Author contribution

Ore Bajomo – study design, data collection, data analysis, writing. Rumneek Hampal – data collection, data analysis. Paul Sykes – Study design, data analysis, writing. Anur Miah – Study design, data analysis, writing.

## Registration of research studies

1.Name of the registry: Research Registry.

2.Unique Identifying number or registration ID: researchregistry6347.

3.Hyperlink to your specific registration (must be publicly accessible and will be checked): https://www.researchregistry.com/browse-the-registry#home/.

## Guarantor

Miss Oreoluwa Bajomo.

## Consent

Consent not required as this paper is a case series with unidentifiable data from a group of patients.

## Provenance and peer review

Not commissioned, externally peer-reviewed.

## Declaration of competing interest

None.

## References

[1] Griffin M., Alderson D., Taylor J., Mealy K. (2020). Intercollegiate General Surgery Guidance on COVID-19. https://www.rcsed.ac.uk/media/564115/infographic-march-27th.pdf.

[2] Veziant J., Bourdel N., Slim K. (2020 Jun). Risks of viral contamination in healthcare professionals during laparoscopy in the Covid-19 pandemic. J. Visceral Surg..

[3] Statistics by Country for Acute Appendicitis - CureResearch.com [Internet]. [cited 2020 Oct 10]. Available from: http://www.cureresearch.com/a/acute_appendicitis/stats-country_printer.htm.

[4] (2013 Aug). Multicentre observational study of performance variation in provision and outcome of emergency appendicectomy. Br. J. Surg..

[5] Solis E., Hameed A., Brown K., Pleass H., Johnston E. (2020 Jul 8). Delayed emergency surgical presentation: impact of corona virus disease ( <scp>COVID‐19)</scp> on <scp>non‐COVID</scp> patients. ANZ J. Surg..

[6] Nepogodiev D., Bhangu A., Glasbey J.C., Li E., Omar O.M., Simoes J.F. (2020 Jul 4). Mortality and pulmonary complications in patients undergoing surgery with perioperative SARS-CoV-2 infection: an international cohort study. Lancet.

[7] Ruffolo C. (2013). Acute appendicitis: what is the gold standard of treatment?. World J. Gastroenterol..

[8] Athanasiou C., Lockwood S., Markides G.A. (2017 Dec 17). Systematic review and meta-analysis of laparoscopic versus open appendicectomy in adults with complicated appendicitis: an update of the literature. World J. Surg..

[9] Quah G.S., Eslick G.D., Cox M.R. (2019 Jul 13). Laparoscopic appendicectomy is superior to open surgery for complicated appendicitis. Surg. Endosc..

[10] Angioni S. (2020). Laparoscopy in the Coronavirus Disease 2019 (COVID-19) Era. https://gynecolsurg.springeropen.com/articles/10.1186/s10397-020-01070-7.

[11] Agha R.A., Sohrabi C., Mathew G., Franchi T., Kerwan A., O'Neill N for the PROCESS Group (2020). The PROCESS 2020 guideline: updating consensus preferred reporting of CasE series in surgery (PROCESS) guidelines. Int. J. Surg..

